# The Role of the Adenosine System on Emotional and Cognitive Disturbances Induced by Ethanol Binge Drinking in the Immature Brain and the Beneficial Effects of Caffeine

**DOI:** 10.3390/ph15111323

**Published:** 2022-10-26

**Authors:** Bruno Gonçalves Pinheiro, Diandra Araújo Luz, Sabrina de Carvalho Cartágenes, Luanna de Melo Pereira Fernandes, Sarah Viana Farias, Natália Harumi Correa Kobayashi, Enéas Andrade Fontes-Júnior, Samira G. Ferreira, Rodrigo A. Cunha, Rui Daniel Prediger, Cristiane do Socorro Ferraz Maia

**Affiliations:** 1 Neurosciences and Cellular Biology Post Graduation Program, Biological Sciences Institute, Federal University of Pará, Belém 66075-110, PA, Brazil; 2 Behavioral and Inflammatory Pharmacology Laboratory, Health Sciences Institute, Pharmacy College, Federal University of Pará, Belém 66075-110, PA, Brazil; 3 Physiological and Morphological Sciences Department, Biological and Health Science Centre, State University of Pará, Belém 66050-540, PA, Brazil; 4 CNC-Center for Neurosciences and Cell Biology, University of Coimbra, 3004-504 Coimbra, Portugal; 5 Faculty of Medicine, University of Coimbra, 3004-531 Coimbra, Portugal; 6 Department of Pharmacology, Centre of Biological Science, Federal University of Santa Catarina, Florianópolis 88040-900, SC, Brazil; 7 Laboratório de Farmacologia da Inflamação e do Comportamento, Instituto de Ciências da Saúde, Universidade Federal do Pará, Rua Augusto Corrêa 1, Belém 66075-900, PA, Brazil

**Keywords:** ethanol, binge drinking, adolescence, adenosine, caffeine

## Abstract

Binge drinking intake is the most common pattern of ethanol consumption by adolescents, which elicits emotional disturbances, mainly anxiety and depressive symptoms, as well as cognitive alterations. Ethanol exposure may act on the adenosine neuromodulation system by increasing adenosine levels, consequently increasing the activation of adenosine receptors in the brain. The adenosine modulation system is involved in the control of mood and memory behavior. However, there is a gap in the knowledge about the exact mechanisms related to ethanol exposure’s hazardous effects on the immature brain (i.e., during adolescence) and the role of the adenosine system thereupon. The present review attempts to provide a comprehensive picture of the role of the adenosinergic system on emotional and cognitive disturbances induced by ethanol during adolescence, exploring the potential benefits of caffeine administration in view of its action as a non-selective antagonist of adenosine receptors.

## 1. Introduction

Ethanol is the most commonly used drug by adolescents, mainly consumed through a binge drinking pattern. According to the National Institute on Alcohol Abuse and Alcoholism (NIAAA), binge drinking consumption is characterized by approximately 0.08% grams of alcohol/dL, which corresponds to the intake of four drinks for women and five drinks for men during 2 h [1]. Evidence from human and laboratory animal studies highlighted the profound structural and functional neurodevelopment processes modifying synaptic plasticity and dendritic connectivity during adolescence [2]. This on-going neuronal maturation predisposes the central nervous system (CNS) to harmful consequences of drugs (i.e., ethanol), eliciting anxiety and depressive symptoms as well as cognitive deficits [3,4,5]. These ethanol-induced behavioral changes in adolescents result from disturbances in homeostasis of several brain regions, such as the prefrontal cortex, hippocampus, and limbic system, which aggravates adolescent risk behavior [6]. In addition, ethanol also negatively affects the mesocorticolimbic pathway, which is part of the reward and reinforcement circuitry. Activation of the dopaminergic system signaling on the ventral tegmental area and nucleus accumbens, concomitant to hyperactivation of the glutamatergic system in limbic structures, trigger neurotoxicity mechanisms and behavioral alterations, especially in the immature brain [7,8].

Our research group demonstrated that the binge drinking paradigm from adolescence until adulthood in animal models induces emotional and motor alterations as well as cognitive deficits related to oxidative damage in several brain areas, such as the hippocampus and prefrontal cortex [9,10,11]. However, we found that the deleterious effects of binge ethanol drinking were not restricted to the central nervous system (CNS). In fact, the stomatognathic system is markedly affected by binge drinking patterns [12]. In this context, we investigated strategies of protection or treatment against ethanol’s hazardous effects on body systems, especially the CNS. For instance, we demonstrated that physical exercise is a useful tool to attenuate or prevent ethanol damage [13]. Moreover, we demonstrated that caffeine is able to prevent ethanol-induced alveolar bone loss in adolescent rats [14]. We further explored the effects of caffeine on the CNS in view of the known beneficial effects of the regular intake of moderate doses of caffeine [15], in particular, to attenuate neurotoxicity in different animal models of brain diseases [16,17].

Although caffeine has multiple molecular targets, it was first proposed by Bertil Fredholm late last century that caffeine mostly acts through the antagonism of adenosine receptors [18]. Indeed, it was recently confirmed that the ability of caffeine to control synaptic transmission and plasticity in hippocampal circuits is critically and solely dependent on the antagonism of adenosine receptors [19]. Adenosine is a prototypical neuromodulator released in an activity-dependent manner, with a parallel role in fine-tuning neuronal function under physiological conditions and controlling neurodegeneration in different neuropsychiatric conditions [20]. Adenosine signals through adenosine receptors, namely A1, A2A, A2B, and A3 [21]. These four metabotropic receptors can recruit numerous transduction pathways, in particular, the formation of intracellular cyclic adenosine monophosphate (cAMP). Adenosine A1 and A3 receptors are coupled to Gi/Go protein, resulting in the inhibition of adenylate cyclase activity and consequent reduction of cAMP formation, whereas A2A and A2B receptors are coupled to Gs proteins, activating adenylate cyclase that increases cAMP production [22].

Adenosine receptors have a wide but heterogenous distribution in the brain. Adenosine A1 receptors (A1R) are the most abundant adenosine receptor subtype, with higher levels in the limbic cortex and thalamus. A1R potently inhibit glutamatergic transmission throughout the brain, as well as dopamine release in corticostriatal neurocircuits [23,24]. On the other hand, adenosine A2A receptors (A2AR) are sparsely but widely distributed throughout the brain to selectively control synaptic plasticity processes [16,25,26], and they are more densely located in the basal ganglia to integrate dopaminergic modulation of corticostriatal glutamatergic transmission [27,28,29]. These adenosine receptors interact with dopamine receptors as A1/D1 and A2A/D2 receptor heterodimers, respectively [30], to efficiently regulate the mesocorticolimbic system and control addiction circuits [31].

The molecular mechanisms associated with drug abuse involve multiple processes ranging from neurotransmitter reuptake blockade, increase in excitatory neurotransmitters release, as well as high extracellular monoamine levels in synapses (reviewed in ref. [32]). Ethanol increases the synaptic levels of adenosine through direct and indirect processes [33,34,35]. Physiologically, the bidirectional equilibrative nucleoside transporters (ENT1) regulate adenosine intracellular and synaptic levels, and ethanol inhibits the activity of ENT1 (a direct mechanism), increasing adenosine levels in the synaptic cleft [36]. Chronic exposure to ethanol triggers neuroadaptations in the densities of A1 and A2A receptors, which may contribute to ethanol abuse and neurotoxicity [34,35,37].

The indirect process is a result of ethanol metabolism to acetaldehyde by alcohol dehydrogenase, CYP2E1 and catalase enzymatic systems. Subsequently, acetaldehyde is converted to acetate, catalyzed by aldehyde dehydrogenase [38]. The acetate produced is recycled to form the neurotransmitter acetylcholine by an active process (i.e., adenosine triphosphate consumption), increasing the levels of intracellular adenosine [38].

Overactivity of the adenosinergic system has been linked to emotional changes in adolescents following withdrawal from high alcohol consumption that persists until adulthood [39]. Therefore, in this review, we explored the ability of caffeine, a non-selective adenosine receptor antagonist, to attenuate or counteract the deleterious effects of ethanol, considering that caffeine affords neuroprotection in different models of neurotoxicity [40,41], attenuating several symptoms of ethanol intoxication such as fatigue, headache, dizziness, weakness, and others [42]. The present review attempts to provide a comprehensive picture of the role of the adenosinergic system on emotional and cognitive disturbances induced by ethanol during adolescence, exploring the potential benefits of caffeine administration and the molecular mechanisms involved. 

## 2. Ethanol versus Adenosine Effects on Anxiety

Ethanol is a drug commonly used in early adolescence, a period where curiosity, novelty, and risk-taking are prevalent [43]. Such early ethanol intake predisposes these adolescent consumers to a higher probability of ethanol abuse or dependence in adulthood since binge drinking leads to an escalating consumption of alcohol, culminating in a heavy drinking pattern of use, aggravating the neurotoxicological effects of ethanol [44,45,46]. Epidemiological studies have demonstrated that binge ethanol drinking induces mood and anxiety disorders in adolescents, either upon daily or episodic consumption [47,48]. Spear [2] reported that ethanol toxicological consequences are intensified among adolescents as a result of modifications in brain maturation and behaviors that are observed in both clinical and experimental studies. 

Reduction and disruption of the integrity of the white matter, as well as a decrease of connectivity between the prefrontal cortex and limbic regions, i.e., mesolimbic and mesocortical pathways mediated by dopamine signaling, have been found following adolescent ethanol exposure [2,49]. These structural and molecular dysfunctions trigger long-lasting anxiety-like behavior in adulthood. Previous studies have indicated that anxiety-like behavior in rodents is present in several animal models involving ethanol consumption, including the development of social anxiety in male rodents [50], anxiogenic effects in elevated plus-maze in adolescent animal exposure to adulthood [10,51], in the light-dark box [52], and open field paradigms [11,13,53]. 

Our group also investigated the impact of heavy chronic ethanol exposure from adolescence to adulthood (6.5 g/kg/day for 55 days) in female rats, which led to neuronal loss in different brain areas, as well as an increase in oxidative stress accompanied by motor, cognitive, and emotional alterations [54,55]. In particular, we focused on behavioral disruptions elicited by binge drinking models (3.0 g/kg/day; 3 days on-4 days off) to mimic a usual pattern of ethanol consumption among teenagers [9,10,13]. Binge ethanol drinking in adolescent female rats triggers an anxiety-like behavior assessed in the elevated plus maze paradigm, which persists upon long-lasting withdrawal of ethanol consumption (14 days) [11]. In these studies, we highlighted the potential mechanisms involved in ethanol’s hazardous effects, including oxidative stress and neuroinflammation [55]. However, additional pathophysiological pathways have also been documented, such as alterations of different neurotransmitter systems, mainly an over-function of the glutamatergic pathway and downregulation of GABAergic signaling [56,57]. Alternative pathophysiological mechanisms underlying the impact of ethanol on anxiety should be further studied to provides a reasonable comprehension of adolescent brain alterations. In this review, we highlight the interaction of ethanol with the adenosinergic system on anxiety-like behavior, mainly during withdrawal.

Some studies suggest that ethanol may increase adenosine levels in the brain by acetate-oxidation (acetyl-CoA to ATP) and inhibition of cellular uptake by ENT-1 blockade [58]. This overactivity of the adenosine system may result in different excitatory mechanisms by alteration of the balance between adenosine A1 (inhibitory) and A2A (excitatory) receptors, consequently affecting other neurotransmitters involved in anxiety [58]. As mentioned above, A1R are widespread in the brain, with the highest expression in the hippocampus, cerebral and cerebellar cortex, and thalamic nuclei [59]. Additionally, A1R are moderately expressed in the caudate-putamen and *nucleus accumbens*, acting presynaptically and postsynaptically [23]. In turn, A2AR have the highest density in basal ganglia and are also present in the extended amygdala and hypothalamus that are involved in the modulation of anxiety and stress [60,61].

The exploration of anxiety-like behavior (elevated plus maze and open field test) at several time points after withdrawal of ethanol intake following an intraperitoneal administration of an acute ethanol dose (4 g/kg) revealed a more pronounced alteration of anxiety between 12–18 h [62]; the acute administration of an A1R agonist (CCPA: 0.05, 0.125, and 0.25 intraperitoneally) reduced of anxiogenic-like behavior in the elevated plus-maze, whereas the administration of the selective A2AR agonist (DPMA) had no effect. Conversely, the selective A1R antagonist 8-cyclopentyl-1,3-dipropylxanthine (DPCPX) triggered anxiety. These findings were also reported by another group [63] using the A1R agonist R-N6-phenylisopropyladenosine (R-PIA) and the A2AR agonist 2-p-(2-carboxethyl) phenylethyl-amino-5′-N-ethylcarboxamidoadenosine (CGS 21680). Other studies also suggest the direct involvement of adenosine on anxiety, since A1R knockout mice displayed increased anxiety and an aggressive profile [64,65]. These results indicate that A1R may be involved in anxiety-like behavior and emerges as a promising pharmacological target to attenuate anxiety conditions [66].

A2AR knockout mice also display alterations of anxiety-like behaviors, and ADORA2A polymorphisms are associated with social behavior and exploratory activity, eliciting anxiety-like behavior with the involvement of the anterior cingulate cortex and amygdala [67,68,69,70]. Accordingly, the genetic deletion of neuronal A2AR prevents stress-induced anxiety [17], whereas the overexpression of A2AR leads to an anxiogenic profile [71]. This also implies a role of A2AR in the control of anxiety [72,73].

We hypothesize that ethanol exposure induces hyperexcitability of the adenosinergic system in the adolescent brain, eliciting two fundamental alterations: (i) disruption of brain maturation, promoting unbalance of adenosine A1/A2A receptors, inducing anxiety behavior, and (ii) modifying adenosine-dependent neurotransmitter levels and the activity of neurocircuits involved in anxiety.

The impact of ethanol intake on the density and expression of adenosine receptors has resulted in somewhat conflicting results. Thus, chronic heavy intermittent ethanol vapor exposure followed by withdrawal (blood ethanol concentration 162.1–217.9 mg/dL) for 64 h, followed by 8 h of withdrawal or not, causes an overexpression of A1R in the cerebral cortex, with no changes of A2AR density in the striatum [74]. In contrast to these findings in adult rodents, the intake of ethanol in adolescent mice triggers a persistent reduction of brain A1R density during withdrawal [39]. A reduction of A1R expression and density in the cerebral cortex and cerebellum of the offspring of dams exposed to ethanol was also observed [75]. Notably, there is a positive correlation between A2AR affinity and the A2AR/A1R affinity ratio but a negative correlation between A1R affinity and the potency (ED50) of adenosine agonists to accentuate ethanol-induced motor incoordination [76]. In general, noxious situations trigger a downregulation of A1R and an upregulation of A2AR [20,71]. 

These adaptive changes are expected to contribute to an increase in excitatory glutamatergic synaptic transmission [77,78], mainly by a reduction of A1R density, impairing inhibitory control in synapses, as reported in experimental and clinical studies [79,80]. In particular, both glutamatergic N-methyl-D-aspartate (NMDA) receptors and voltage-sensitive calcium channels are controlled by the tonic activation of A1R [81,82], as well as by A2AR [83], implying that ethanol can imbalance the control of synaptic plasticity as well as of neurodegeneration that is critically dependent on NMDA receptors and voltage-sensitive calcium channels [84].

Apart from this imbalanced adenosine modulation of plasticity that is critical for the development of additive behaviors, adenosine modulation of reward circuitry is also altered [32,72,85]. Reward circuitry activation by glutamatergic inputs from the cortex, as well as dopaminergic inputs from the ventral tegmental area with projections to medium spiny neuron striatum, through heterodimers of A2A-D2 and A2A-mGlu5 receptors, may be probable pathophysiological mechanisms induced by ethanol abuse since this substance increases adenosine levels causing hyperactivation of A2AR, with consequent increased release of dopamine and glutamate [32,86,87]. Consequently, neural excitotoxicity, changes in homeostatic regulation by oxidative stress, abuse risk, and several behavioral alterations, such as anxiety, occur [35].

Adenosine receptors, in particular A2AR, control the activity of the hypothalamus–pituitary–adrenal (HPA) axis [88]. In particular, adenosine modulates different circuits of the pituitary gland [89]. In the intermediate region, the blockade of A2AR reduces proopiomelanocortin and alfa-MSH levels, reducing the activation of the HPA axis [90]. Conversely, the inhibition of A2AR in the anterior lobe of the pituitary hyperactivates the HPA axis, increasing proopiomelanocortin, adrenocorticotropic hormone, and consequently blood corticosterone levels [90], which characterizes the anxiety-related profile. However, further investigations focused on ethanol-induced anxiety versus adenosinergic modulation of the HPA axis during adolescence should be undertake. 

In summary, the knowledge of the balance between adenosine receptors (A1 and A2A) in the adolescent brain and the control of neurotransmitters in different neurocircuits is a significative step toward elucidating our hypothesis. Such well-outlined mechanisms may support critical strategies for neuroprotection or treatment of anxiety induced by ethanol consumption in adolescents by pharmacological or genetic manipulations targeting adenosine receptors.

## 3. Ethanol versus Adenosine Effects on Depression

Depression is an affective disorder characterized by the presence of mood dysregulation typified by a depressed mood (dysphoria) and reduced ability to have pleasure (anhedonia). Depressed patients may also present cognitive impairment and somatic symptoms, leading to significant distress or impairment in general body system functioning [91,92,93]. Depressive disorders can be triggered by several etiologies, including drug abuse, such as opioids, sedatives, stimulants, and hallucinogens, whereas depressive symptoms can appear during or shortly after intoxication or discontinuation of the drug of abuse [91,94,95,96]. 

Epidemiological studies have consistently concluded that alcohol intake in a binge pattern, mainly in late adolescence, elevates the risk of developing depressive symptoms in young women between 20 to 30 years of age, when the consumption occurs frequently, approximately 16% [97]. Moreover, drinking habits are often associated with depressive symptoms and suicide in young individuals, with circa 11.5% showing depressive behavioral and 2.8% suicidal ideation [98]. Ethanol is a CNS depressant which triggers depressive symptoms by different molecular targets. According to Alasmari et al. [99], ethanol consumption elicits modifications in dopamine, glutamate, and GABA neurotransmitter release. It is noteworthy that significant dopaminergic reductions in the reward system or in neurotransmitter recruitment play a role in the progression of negative reinforcement, resulting in psychoneuroimmunological neuroadaptations related to neuroinflammation and emotional disruption [99,100,101,102]. It has also been reported that ethanol exposure reduces brain-derived neurotrophic factor (BDNF) in the hippocampus [103,104,105]. Such alterations are more harmful during adolescence since, during brain maturation, an unbalance of neuromodulatory mediators affects limbic circuitry, impairing the development of neurocircuitry in the prefrontal cortex, leading to increased limbic reactivity and consequently changes in affective control [106,107].

In addition, adolescent subjects present elevated amygdala activity and decreased fear extinction, mediated by changes in prefrontal cortex–amygdala connectivity [108]. Furthermore, the adolescent brain is particularly sensitive to repeated ethanol exposure. Thus, ethanol neurotoxicity associated with enhanced emotional reactivity and poor effective control displays augmented risk of emergence and exacerbation of emotional dysregulation, such as depression [2,107,109].

Accordingly, our group has reported, using animal models, that ethanol exposure during brain development elicits a depressive profile, even after long-term abstinence [9,110], with a reduction in BDNF levels in the hippocampus immediately upon withdrawal [10]. This observation is particularly relevant since there is a strong relationship between the negative effects displayed by ethanol in neurotransmitter homeostasis, the HPA axis, and neurotrophic factors [111]. However, alternative pathophysiological mechanisms may explain the depressive profile elicited by ethanol intake in adolescent CNS, and one such mechanism involves a dysfunction of the adenosine modulation system.

An interesting study indicated a relationship between adenosine and the pathophysiology of alcoholism and depression [112]. Inhibitory mechanisms of adenosine in the CNS, which modulate excitability, neurotransmitter release, and ion channel function regulation, play a role in mood changes in alcohol-exposed patients [23,113,114]. In cell culture assays, ethanol acute exposure increases adenosine levels and contributes to intoxicating and/or rewarding effects [115,116]. High levels of adenosine hyperactivate A2AR signaling, which develops desensitization across prolonged ethanol exposure [117]. Another fundamental neuroadaptation consists of the reduction of the plasma membrane nucleoside transporter ENT-1, which results in reduced extracellular and synaptic adenosine levels [72,117]. Despite these findings, few studies have addressed the impact of alcohol exposure during adolescence on the adenosine modulation system.

Scarce studies have demonstrated that repeated ethanol administration (2.0 g/kg) in adolescent mice increased the binding activity of cAMP response element-binding protein (CREB) in the prefrontal cortex and hippocampus [118]. It is well-defined that elevation of CREB expression in the dorsomedial striatum, olfactory bulb, and GABAergic neurons of caudate-putamen, *nucleus accumbens*, and tuberculum olfactory, also occurs upon recruitment of A2AR and is likely associated with negative behavioral changes (i.e., anxiety-like and depressive-like phenotype) induced by heavy ethanol consumption in mice [71,119]. 

Taken together, the available evidence is suggestive of the involvement of the adenosine modulation system in the depressive-like profile induced by ethanol exposure during adolescence, namely through CREB overexpression resulting from the overactivation of A2AR. It is noteworthy that A2AR hyperactivation directly influences A2A/D2 heterodimerization, as already mentioned above when discussing anxiety [120,121]. Accordingly, functional interrelationships related to mesocortical and mesolimbic pathways of A2A/D2 receptor interactions that are impaired by ethanol administrations may result in emotional, motivational, rewarding, and addiction behavior disruption and learning dysfunction, which reinforces the putative role of the adenosine modulation system in several neuropathologies, such as anxiety, drug addiction, schizophrenia, and depression [71,122].

To support this link between A2AR modulation and depressive-like behavior through the influence of dopamine levels, Coelho et al. [71] investigated the impact of A2AR overexpression in cortical areas for dopamine-related behavior. These authors found that the hyperactivity of the A2AR pathway induces a depressive-like phenotype [71,123,124]. Furthermore, Kaster et al. [17] reported that the chronic caffeine administration or selective adenosine A2AR antagonism or genetic deletion of adenosine A2AR is able to prevent or revert mood and memory dysfunction, as well as neurochemical and synaptic deficits induced by chronic stress.

In summary, acute and/or chronic ethanol exposure during adolescence disturbs the homeostasis of the adenosine modulation system in the brain, contributing to hazardous symptoms related to depression. In addition, overexpression of A2A/D2 receptors in mesocorticolimbic areas, preferably in the forebrain, has been associated with depression behavior, which may explain the depressive signs seen in aging and chronic stress [71]. 

## 4. Ethanol versus Adenosine Effects on Cognition

Cognitive functioning depends on multiple integrated processes occurring in distinct areas of the CNS. For instance, the acquisition of declarative (or spatial) memories begins in the hippocampus, through synaptic changes, since damages to this structure compromise recent memory, while remote memories remain intact. This fact suggests that cognitive storage occurs in other structures, such as the neocortex, which has been widely pointed out as an important storage location [125,126,127]. In turn, the targeting/selection of memories that will become long-lasting is regulated by environmental factors and emotionality, among other factors, and this modulation is operated by structures such as the prefrontal cortex amongst others [126,128].

Classically, the neurotransmitters glutamate and acetylcholine play a fundamental role in memory processing [129,130]. Nonetheless, other signaling systems robustly regulate memory acquisition, including the adenosine modulation system. Imbalances in the adenosine system affect several CNS functions, including cognition, whereas overactivation of adenosinergic receptors, especially the A1R and A2AR subtypes, elicit memory impairment [121]. Although it is complex to define the exact contribution of the different adenosine receptors to the control of cognition since their responses differ upon homeostatic or pathological conditions [131,132,133], a prominent role of A2AR seems evident: this is best heralded by the observation that the pharmacological overactivation of A2AR [134] or the overexpression of A2AR in forebrain neurons [135] or the opto-stimulation of the A2AR transducing system [136] are each sufficient to cause a disruption of spatial reference memory performance.

In keeping with our hypothesis of a parallel an opposite deregulation of the A1R/A2AR imbalance upon repeated ethanol intake, we propose that cognitive deficits may also be dependent on A1R/A2AR activity. Thus, overactivation of A1R inhibits the release of glutamate and acetylcholine, impairing cognition processes, such as memory acquisition and consolidation mediated by the hippocampus [129,130]. The overactivity of A1R may lead to cognitive impairment. Accordingly, acute treatment with micromolar doses of A1 receptor agonists induced deficits in memory acquisition and retention, whereas the administration of selective A1 receptor antagonists reversed these negative effects [137]. 

Therefore, substances that promote an increase or imbalance in adenosine receptor activity may produce mnemonic impairments, especially in critical periods of development/remodeling of the CNS [138]. Epidemiological data reveal that ethanol consumption, especially in a binge pattern, usually starts during adolescence [138,139,140,141], and neural circuits in the immature brain are vulnerable to several factors that modulate brain function [141]. 

Accordingly, we reported that the cumulative four cycles of binge drinking paradigm (3 g/kg/day) during adolescence impairs short-term memory in object recognition tasks in the immediate ethanol withdrawal period [10]. In agreement with this, other binge drinking studies during adolescence also found mnemonic disruption by applying diverse cognitive tests [8,142,143,144], highlighting the potentially hazardous effects of binge-like consumption on distinct types of memory.

Numerous pathophysiological mechanisms have been attributed to mnemonic abnormalities. Oxidative stress, deficits of neurotrophin levels, glutamatergic hyperactivity, and reduction of neuronal viability and survival have been considered as possible causes of memory impairments induced by adolescent alcohol binge drinking [8,142,143,144,145]. Although all these previously described mechanisms induce mnemonic disturbances, the probable involvement of the adenosine system should also be considered. Indeed, it was reported that the acetate originating from ethanol metabolism could be incorporated into acetyl-coenzyme A, supporting the production of cAMP and adenosine, thus bolstering adenosinergic signaling [146]. In addition, alcohol consumption also inhibits adenosine reuptake, which increases the extracellular levels of adenosine and, consequently, its actions [72]. These effects likely depend on the pattern of alcohol exposure. Acutely, alcohol increases adenosine levels, which leads particularly to sedation and cognitive impairment [137]. Chronic exposure seems to trigger a reduction of ENT-1 expression and an influx of adenosine, as mentioned above [115,146]. Both responses impair the balance of influx/efflux of adenosine, thus reducing its regulatory activity, a reduction further aggravated by the early heterologous desensitization of A1R and A2AR. Microdialysis studies detected a four-fold increase in adenosine levels in the brain parenchyma following ethanol exposure, which, among other responses, contributes to its sedative/hypnotic properties, in addition to inducing cognitive disorders [137]. In fact, animal and human studies confirm the potential of ethanol to display memory impairment related to adenosine overactivity. Obviously, these toxicological events can also occur in adolescents and adult individuals. Studies in zebrafish exploring the long-term consequences of early ethanol exposure in distinct embryonic stages indicated the emergence of a mnemonic impairment, which was reversed by acute administration of an ecto-5’-nucleotidase inhibitor (an enzyme that converts extracellular AMP into adenosine) [147]. This emphasizes the influence of the adenosine system on persistent cognitive deficits induced by ethanol exposure during neurodevelopment [147]. 

However, there are some peculiarities related to maturing processes during adolescence, which might elicit different results. For example, both increased expression of adenosine receptors and downregulation of their reuptake seem to be associated with continuous consumption, accompanied by multiple episodes of withdrawal [37,115]. This fact is of relevance since the binge drinking, frequently performed by teenagers, is characterized by an intermittent consumption, which provides favorable conditions for the occurrence of these mechanisms [139,141]. Unfortunately, few approaches have assessed the relationship of this pattern of alcohol intake with adaptations of the adenosine system affecting memory processing, especially during adolescence, which await further investigations to unravel novel therapeutic strategies. Table 1 summarizes the studies addressing the involvement of the adenosine system in the behavioral and cognitive impairments induced by ethanol.

## 5. Caffeine as a Therapeutic Tool in Ethanol-Induced Anxiety, Depression, and Cognitive Disorders

### 5.1. Anxiety

The hypothesis that the toxicological mechanisms of ethanol exposure result from hyperexcitability of both A1R and A2AR function entails the conclusion that a non-selective antagonism of A1R and A2AR, such as that afforded by caffeine [17,18], may be particularly effective to manage the behavioral disturbances caused by exposure to ethanol. We next discuss: (i) whether the non-selective blockade of A1R/A2AR should be considered a potential target to revert the anxiety profile induced by ethanol; (ii) if the partial inhibition of adenosine receptors induces or normalizes the balance and tonus during brain maturation of adolescents; (iii) what dose and time regimen of caffeine intake would be necessary to afford neuroadaptive benefits? 

Caffeine emerges as a useful nutraceutical tool since this bioactive compound is a non-selective adenosine receptor antagonist that is generally profiled to manage anxiety disorders. The acute intake of caffeine triggers anxiogenic effects in humans and can bolster panic attacks; tolerance emerges with continued administration and anxiety also emerges upon withdrawal. Experimental studies demonstrate that caffeine (25 and 50 mg/kg, intraperitoneally) display anxiogenic-like effects in the elevated plus-maze paradigm, whereas it has no effect at 10 mg/kg; This suggests that caffeine presents contradictory effects depending on distinct variables, such as dose regimen [148,149,150]. According to Fredholm [151], non-toxic doses caffeine selectively blocks different subtypes of adenosine receptors. Such low doses of caffeine are equivalent to the intake of 1-3 cups of coffee, which decrease the actions of adenosine receptors, conferring a beneficial treatment or protection strategy. Thus, we assume that the modulation of anxiogenic repertoire by caffeine depends on the dose, the pattern of administration, gender, age, and period of exposure [152]. However, the relationship between caffeine dose versus anxiety-like phenotype is still contradictory [148,153,154]. Firstly, acute caffeine at a high dose (25–50 mg/kg) can elicit opposite anxiogenic and anxiolytic effects in behavioral tasks [148,153,154]. Such differences have been attributed to different anxiogenic levels intrinsically related to different animal strains [154]. Furthermore, repeated exposure to caffeine as well as noxious stimuli lead to an adaptative alteration of the expression and density of adenosine receptors in the brain, which pave the way to consider that chronic administration of low caffeine doses may be an important tool to revert or attenuate the anxiogenesis in pathological conditions, namely upon exposure to ethanol. 

We established and validated a new protocol of chronic caffeine treatment (10 mg/kg for 21 days) in adolescent female animals submitted to a binge drinking ethanol challenge. Firstly, we investigated alveolar bone homeostasis, in which caffeine prevented ethanol bone loss that was not mediated by A2AR blockade [14]. Presently, we are testing this protocol in an anxiety-like model, assessing new strategies of pharmacological manipulations as therapeutic tools, i.e., agonism of A1R and antagonism of A2AR. An important study conducted by Prediger et al. [62] demonstrated that A1R agonism in an ethanol hangover model elicits anxiolytic effects, inferring that the downregulation blockade and consequent inhibition of glutamate release are the main mechanisms related to anxiolysis. However, A2AR should not be neglected since it has been indicated as a potential strategy of neuroprotection in several models of brain damage [155,156,157]. We anticipate that the blockade of A2AR may rebalance the abnormal release of glutamate and catecholamines involved in anxiety. 

### 5.2. Depression

As previously mentioned, caffeine may present potential pharmacological features to attenuate depressive-like behavior displayed by ethanol exposure. Different studies have reported that patients suffering from psychiatric disorders tend to increase caffeine intake and longitudinal and prospective studies indicate that caffeine consumption decreases the risk of depression and suicide [158,159,160].

The inverse relationship between caffeine intake and mood deterioration was further confirmed in animal studies, where the prophylactic administration of caffeine (1 g/L) prevented mood and memory alteration induced by chronic stress [17]. Furthermore, the authors identified that neuronal A2AR play a critical role in controlling chronic stress induced in mice and may reverse changes caused by repeated stress [17]. A similar conclusion was obtained in stressed or depressed individuals [31,161] and in different animal models [73], reinforcing the inverse relationship between caffeine consumption and the incidence of depression [162,163] or suicide [164,165].

It is noteworthy that further research is fundamental since contradictory findings have been reported: moderate doses of caffeine (7–8 mg/kg) added to ethanol beverages in a self-administration protocol elicited a depressive-like phenotype in a sex-dependent manner, positive for female rodents [10]. In addition to these experimental findings, clinical studies reported depressive symptoms following withdrawal after intake of moderate doses of caffeine (235 or 600 mg/day), suggesting an abstinence syndrome adverse effect [166,167]. However, these studies focused on withdrawal symptoms following caffeine-induced dependence [168].

Preclinical studies involving alcohol, depression, and caffeine are still scarce. However, there is evidence that alcohol-induced depressive behavior may be attenuated by pre- or post-administration of caffeine. However, the regimen of alcohol and caffeine protocols has resulted in conflicting results with respect to the effects of caffeine on alcohol-induced depression. Clearly, additional studies are necessary to clarify this issue.

### 5.3. Cognitive Deficits

There is substantial evidence that the regular consumption of moderate caffeine attenuates memory deficits [169]. This is most evident when considering the beneficial effects of caffeine intake in different models of Alzheimer’s disease such as in transgenic models leading to the over-function of the amyloid cascade [170,171] or tau [172], as well as in models of sporadic Alzheimer’s disease [161]. This prevention of memory deficits by caffeine is mimicked by the pharmacological or genetic blockade of A2AR [133,135,172,173,174]. Moreover, caffeine also prevents memory impairment in different animal models of neuropsychiatric disorders through the antagonism of A2AR [175,176,177]. This paves the way for an expected benefit associated with caffeine intake to dampen ethanol-induced memory impairment. 

The interaction between ethanol and caffeine treatment has been studied under two contexts: concomitant use in a recreational context and as a therapeutical tool to prevent or reverse cognitive disturbances induced by alcohol. Here, we have chosen the therapeutical context to describe the therapeutic aspect of this association. Human studies demonstrated that the chronic consumption of caffeinated beverages improves reaction time (an attentional, probed-recall memory performance, and cognitive measurement) in healthy volunteers chronically administered with low doses of ethanol challenge (0.5–0.75 g/kg) [178,179]. Such clinical findings are in line with experimental studies reporting that an acute dose of caffeine (50 mg/L) improves memory deficits induced by chronic ethanol exposure in zebrafish models, whereas a higher caffeine dose (100 mg/L) failed to improve ethanol-induced cognitive deficits [180]. Additionally, the previous administration of low doses of caffeine (5 mg/kg) prevents retrograde memory damage induced by a single dose of ethanol (3.0 g/kg) [181]. All these results indicate that caffeine might be an effective therapeutic tool to prevent or mitigate alcohol-induced mnemonic disorders, although it still remains to be defined which adenosine receptor is involved in this caffeine-mediated alleviation of ethanol-induced memory impairments.

Thus, we provide fertile grounds to plough the hypothesis that caffeine might be a novel, potentially relevant strategy to attenuate some of the deleterious effects of ethanol. However, these putative beneficial effects likely depend on several interfering factors [10,34], and several mechanistic questions remain unresolved. Table 2 summarizes the studies addressing the effects of caffeine on anxiety, depression, and cognitive impairments induced by ethanol.

**Figure 1:**
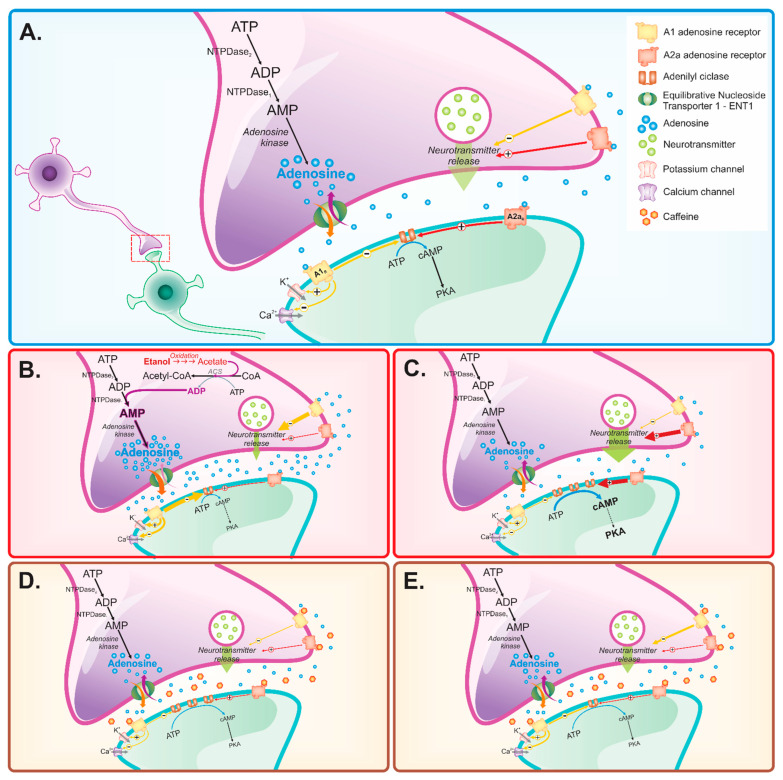
Adenosine action on A1 and A2A receptors promotes important regulation of neurotransmission in the brain (**A**); excessive consumption of ethanol has the potential to raise adenosine levels, modulating ENT1, generating increased levels and hyperactivity of adenosine in the synaptic cleft, especially acting on A1 receptors (**B**); as a consequence, neuroadaptation occurs, reducing the tone of A1 receptors, favoring the hyperactivity of A2A receptors (**C**). Caffeine, by blocking adenosine receptors, promote the modulation of A2A receptor-mediated hyperactivity, reducing the effects resulting from ethanol consumption (**D**), and, in the long term, could reverse neuroadaptation (**E**). Thus, in theory, caffeine could be used as a therapeutic agent to combat the deleterious effects of ethanol.

The contradictory evidence related to co-exposure versus beneficial/detrimental effects probably results from the lack of well-designed investigations to isolate the different variables. We also highlight the putative potential of binge drinking during adolescence to imbalance the adenosine modulation system, which still requires further investigation. If such neuroadaptations are confirmed on cognitive impairment, is caffeine able to attenuate or prevent this dysfunction? Under what conditions (dose, frequency, and consumption time) do these benefits occur? These are some questions in this field that still need to be answered. Figure 1 shows the probable targets related to caffeine in preventing brain alterations induced by ethanol on emotionality and cognition impairment.

## 6. Conclusions

Ethanol is a psychoactive substance widely consumed by young individuals. It is well documented that ethanol consumption elicits several negative effects on emotionality and cognitive function, which might persist into adult life, suggesting different neurotoxicological mechanisms according to the pattern of consumption. Few studies have addressed the molecular processes involving the adenosine modulation system in the behavioral changes induced by ethanol consumption, especially in a binge-drinking pattern. In this review, we highlighted some probable events triggered by ethanol exposure to mediate its harmful effects focusing on the alteration of the adenosine neuromodulation system during adolescence and persisting until adulthood. In addition, we presented a critical discussion about the unbalance of adenosine A1/A2A receptors to justify the role of caffeine (a non-selective blocker of adenosine receptors) at low doses as a robust neuroprotection strategy for improving emotional disorders and cognitive impairments induced by ethanol exposure.

## Figures and Tables

**Table 1:** The involvement of the adenosinergic system in behavioral and cognitive impairments induced by ethanol.

Drug(s)	Evaluation Condition	Study Information	Tests and Analysis	Main Effects	Development Period and Possible Mechanisms	Reference
Ethanol DPCPX-selective adenosine A_1_ receptor antagonist (CCPA)-selective adenosine A_1_ receptor agonist	Under drug withdrawal	Pattern of use: acute withdrawal Type: pre-clinical study Dose and use frequency: 0.05 mg/kg i.p. of CCPA 15 min before of 3 mg/kg i.p of DPCPX in ethanol withdrawal of 18 h in the dose of 4 g/kg i.p.	Open field and Elevated Plus Maze test during hangover	The anxiogenic effect of CCPA was reverted within 18h of withdrawal	Adult mice/agonism of A1R and antagonism of A1R supporting the involvement of A1R	[62]
Ethanol	Under drug withdrawal	Pattern of use: acute and chronic withdrawal Type: pre-clinical study Dose and use frequency: ethanol 1.6 g/kg (8% *w*/*v*) by inhalation in four cycles of 16 h followed by 8 h of abstinence; acutely (single withdrawal in 16 h) and chronically (multiple withdrawal in 64 h)	Effects of single and repeated episodes of ethanol withdrawal on A1R and A2AR in controlling ethanol-induced convulsions	Increase in the convulsion score upon ethanol withdrawal	Adult mice/higher expression of A1R in the cortex	[74]
Ethanol	Under drug withdrawal	Pattern of use: chronic withdrawal Type: pre-clinical study Dose and use frequency: administration of ethanol-free liquid diet (3.5% *w*/*v*) with discontinuation during 6 h after 18 days	Withdrawal score and relative expression and density of NMDA, AMPA, A1R, and A2AR	Increase in seizures, hyperreflexia, and running episodes	Early adolescence to adulthood/higher expression of NMDA and AMPA, reduction of A1R, and no alterations of A2AR	[39]
Ethanol	Under drug effect	Pattern of use: chronic exposition Type: pre-clinical study Dose and use frequency: administration of ethanol in water (15% *v*/*v*) during fetal phase in female rats, and after 60 days, the offspring was tested	Body and brain weights, as well A1R expression in cortex, cerebellum, hippocampus and striatum	Reduction in weight and lower expression of A1R in cortex and cerebellum	Fetal development and offspring/ reduction of A1R	[75]
Ethanol	Under drug withdrawal	Pattern of use: acute withdrawal Type: pre-clinical study Dose and use frequency: administration of ethanol (6.7% *v*/*v*) with discontinuation in 6–7 h	Withdrawal score	Increase of irritability	Late adolescence until adulthood/roleof the adenosine receptors; higher expression acutely of nucleoside transporters	[63]
Ethanol CGS21680-selective adenosine A_2A_ receptor agonist	Under drug withdrawal	Pattern of use: acute withdrawal Type: pre-clinical study Dose and use frequency: 0.3 mg/kg i.p, during 6 h (0.5 h withdrawal) to 7 h (1.5 h withdrawal)	Withdrawal score	Reduction of irritability	Late adolescence until adulthood/agonism of A1R and A2AR with high expression of adenosine transporters in striatum	[63]
Ethanol	Not informed	Pattern of use: chronic exposition Type: pre-clinical study Dose and use frequency: The concentration of ethanol was raised every fourth day, increasing from 3 to 5 to 10% (*v*/*v*) for 10 weeks	Forced swim test, open field and marble-burying test	Anxiogenic and depressive behavior	Adult/ENT1 null mice have lower adenosine levels in the striatum and reduced A1R activation	[112]
Ethanol Adenosine	Not informed	Literature review	Not informed	Not informed	Changes of adenosine formation, adenosine uptake, and effects on adenosine receptor coupling	[23]
Ethanol Adenosine	Under drug effect	Literature review	Pre-clinical	Ataxia, sleep effects	Not mentioned/relevance of the inhibition of alcohol-sensitive ENT1 in the behavioral effects of ethanol	[113]
Ethanol Adenosine	Withdrawal drug effect	Literature review	Pre-clinical	____________	Acute ethanol increases extracellular adenosine in cultured cells by selectively inhibiting ENT1	[72]
Ethanol Adenosine	Withdrawal drug effect	Pattern of use: chronic exposure Type: in vitro study Dose and use frequency: ethanol: 100 mM for 2 weeks and adenosine 1.5 units/mL for 48 h	High pressure liquid chromatography	____________	Ethanol enhances extracellular adenosine levels in NG108-15 and S49 lymphoma cells, causing increase intracellular cAMP levels mediated by adenosine receptors	[115]
Ethanol	Self-administration	Pattern of use: Self-administration Type: pre-clinical study Finality of use: dependence model Dose and use frequency: ethanol: 3–6 to 10% (*v*/*v*) for 4 days	Two-bottle choice	Goal-directed behavior, density of A2AR in the Dorsomedial Striatum (DMS) and CREB activity	Adult mice/habitual seeking of ethanol is regulated by ENT1; A2AR in DMS regulate ethanol drinking and CREB levels	[72]
Ethanol	Under drug effects	Pattern of use: ethanol acute Type: in vitro study Dose and use frequency: Pretreated with S-(4-nitrobenzyl)-6-thioinosine (100 µM: NBTI); concentrations of ethanol of 0, 25, 50, 100, and 200 mM	Human bronchial epithelial cell line	-------------	EtOH acutely inhibits adenosine uptake via nucleoside transporters and chronic EtOH exposure desensitizes adenosine transporters	[146]
Ethanol AMPCP-inhibitor of ecto-5′-nucleotidase EHNA-inhibitor of adenosine deaminase	Long-lasting effects of ethanol	Pattern of use: chronic withdrawal Type: pre-clinical study Dose and use frequency: Embryos of zebrafish were exposed to 1% (*v*/*v*) ethanol; AMPCP at 150 mg/kg or EHNA at 100 mg/kg i.p. using adult fishes	After 30 min of AMPCP, EHNA injections locomotor anxiety, aggressive and social interaction behaviors were evaluated	AMPCP during the adult phase reversed aggressive parameters, and both inhibitors (AMPCP and EHNA) recovered social interaction	Adult/ecto-5′- nucleotidase and adenosine deaminase activities modulate long-lasting ethanol effects	[147]

**Table 2:** Caffeine, adenosinergic system, and behavior.

Evaluation Condition	Study Information	Tests and Analysis	Behavioral effects	Development Period and Possible Mechanisms	Reference
Under drug effect/abstinence	Pattern of use: acute, subchronic, and withdrawal Type: pre-clinical study Dose and use frequency: Under drug effect: 10, 25, 50, and 100 mg/kg i.p.; followed by 50 mg/kg i.p. for 7, 14, and 21 days. Upon abstinence: 50 mg/kg i.p. for 21 days following 2 days of abstinence	Open Field test, Elevated Plus Maze, and Social interaction test	Low doses cause no alterations/moderate to high doses are anxiogenic	Adult/antagonism of adenosine receptors, noradrenaline transmission, and benzodiazepine ligands	[148]
Several	Pattern of use: acute, chronic, toxic, and withdrawal Type: clinical studies Dose and use frequency: not found	Clinical data	Anxiogenic	Adult/not investigated	[149]
Under drug effect/abstinence	Literature review	Clinical data	Anxiogenic (panic attack at an high dose of 750 mg) under effect/anxiolytic or anxiogenic upon withdrawal	Not mentioned/antagonism of adenosine and noradrenaline overactivity	[150]
Under drug Effect	Pattern of use: chronic Type: pre-clinical and in vitro studies Dose and use frequency: 20 mg/kg i.p. after one week	Cortical slice	Increases the binding density of the A1R ligand [3H]L-phenyl-isopropyl-adenosine	Adult/upregulation of adenosine receptors	[151]
Under drug effects	Pattern of use: acute and pre-treatment Type: pre-clinical and clinical study Dose and use frequency: 8, 15, 30 and 60 mg/kg	Elevated Plus Maze test	Low doses (not alterations) and high doses (anxiogenic)	Adult/adenosine receptors	[152]
Under drug effect	Pattern of use: acute Type: pre-clinical study Dose and use frequency: Under drug effect: 10 and 30 mg/kg i.p.	Elevated Plus Maze	Dose-response curve obtained in a light environment	Adult/participation of the GABAergic pathway	[153]
Under drug effect	Pattern of use: acute Type: pre-clinical study; both gender Dose and use frequency: 25 or 50 mg/kg i.p. during one week	Open Field and Elevated Plus Maze	Anxiolytic	Adult/antagonism of A2AR	[154]
Under drug effects	Pattern of use: acute Type: pre-clinical study Dose and use frequency: 10 or 30 mg/kg i.p.	Elevated Plus Maze test	High doses (anxiogenic)	Agonism and antagonism of adenosine receptors/involvement of A_1_R	[62]
Withdrawal drug effects	Pattern of use: chronic Type: pre-clinical study Dose and use: 1g/L for 3 weeks frequency: ad libitum	Forced-swimming test, tail-suspension test and elevated plus maze	Depressive, anxiogenic, and anhedonia-like behavior	Adult mice/blockade of A2AR	[17]
Withdrawal drug effects	Pattern of use: chronic Type: pre-clinical study Dose and use: 1 g/L for 2 weeks frequency: ad libitum	Open field, novel object recognition task, expression of receptors	Prevented memory impairment and neurodegeneration	Adult/not specified	[161]
Withdrawal drug effects	Pattern of use: chronic Type: pre-clinical study Dose and use frequency: 0.1, 0.3 or 1.0 g/L. Frequency: ad libitum	Open field, Novel object recognition task and elevated plus maze	Anxiogenic behavior, negative impacts on non-associative learning	Adolescence to young adult/adenosine antagonism; neuroinflammation and BDNF	[162]
Withdrawal drug effects	Pattern of use: chronic exposition Type: clinical study Dose and use frequency: coffee regular consumption (235 and 600 mg/day) twice within one week	Withdrawal symptoms	Headache, increased irritability, decreased performance, and disturbed concentration, as well as depression and anxiety	Adolescents and adults/mood impairments such as depression and anxiety	[166,167]
Under drug effects	Pattern of use: acute, subchronic, chronic exposure Type: pre-clinical study Dose and use frequency: several administrations	Alzheimer’s model	Prevention of cognitive decline	Adult mice and cell cultures/neuroprotection by caffeine	[169,170,171]
Under drug effect	Pattern of use: chronic Type: clinical study Dose and use: caffeine (300 mg/day) and ethanol (0.5 g/kg) caffeine (150 mg/kg and ethanol 0.5 g/kg) frequency: not specified	Probed-recall memory, sleepiness scale, memory and profile of mood states	Caffeine reversed the effects of ethanol on reaction time in a dose-related manner	Young adult/not investigated	[178]
Under drug effect	Pattern of use: chronic Type: clinical study Dose and use frequency: caffeine (300 mg/70 kg) and ethanol (0.75 g/kg) o.r.. for 8 weeks	Standing steadiness, auditory, visual and complex reaction time, manual dexterity, numerical reasoning, perceptual speed, verbal fluency	Body sway in up to 40 min; caffeine reduces simple auditory and complex reaction time	Young adult/caffeine antagonized the ethanol-induced increase in simple auditing, simple visual, and complex reaction time	[179]
Withdrawal drug effects	Pattern of use: acute and chronic in combination with ethanol Type: pre-clinical study Dose and use frequency: 0.5% of ethanol and 50 mg/mL of caffeine during 1 day to 15 days	Object discrimination	Learning in the zebrafish model	Adult/combination in withdrawal cause no alterations; low to moderate doses of the combination alter object discrimination	[180]
Under drug effects	Pattern of use: acute ethanol Type: pre-clinical study Dose and use frequency: 0.5% of ethanol and 50 mg/mL of caffeine during 1 day to 15 days	Novel odor	Prevention of retrograde amnesia	Adult rats/low doses of caffeine prevent impairments cognitive induced by ethanol	[181]

## Data Availability

Data sharing not applicable.
